# Classification of Hepatitis Viruses from Sequencing Chromatograms Using Multiscale Permutation Entropy and Support Vector Machines

**DOI:** 10.3390/e21121149

**Published:** 2019-11-25

**Authors:** Ersoy Öz, Öyküm Esra Aşkın

**Affiliations:** Department of Statistics, Yildiz Technical University, 34220 Istanbul, Turkey; oeyigit@yildiz.edu.tr

**Keywords:** hepatitis nucleic acid sequencing, permutation entropy, multiscale permutation entropy, support vector machines

## Abstract

Classifying nucleic acid trace files is an important issue in molecular biology researches. For the purpose of obtaining better classification performance, the question of which features are used and what classifier is implemented to best represent the properties of nucleic acid trace files plays a vital role. In this study, different feature extraction methods based on statistical and entropy theory are utilized to discriminate deoxyribonucleic acid chromatograms, and distinguishing their signals visually is almost impossible. Extracted features are used as the input feature set for the classifiers of Support Vector Machines (SVM) with different kernel functions. The proposed framework is applied to a total number of 200 hepatitis nucleic acid trace files which consist of Hepatitis B Virus (HBV) and Hepatitis C Virus (HCV). While the use of statistical-based feature extraction methods allows representing the properties of hepatitis nucleic acid trace files with descriptive measures such as mean, median and standard deviation, entropy-based feature extraction methods including permutation entropy and multiscale permutation entropy enable quantifying the complexity of these files. The results indicate that using statistical and entropy-based features produces exceptionally high performances in terms of accuracies (reached at nearly 99%) in classifying HBV and HCV.

## 1. Introduction

Investigating sequencing of nucleotides from deoxyribonucleic acid (DNA) and ribonucleic acid (RNA) is an important research area in the field of molecular genetics. Although next-generation sequencing platforms have been getting more applicable than capiller electrophoresis recently, capiller electrophoresis studies are required for the verification of next generation sequencing results. Since assessing the huge number of subjects is time-consuming and cost-intensive, it is widely used in small sized projects. In order to determine the sequencing of interested nucleic acid (DNA-RNA) regions, millions of copies are amplified with the process named polymerase chain reaction (PCR). In PCR, the interested RNA region is also converted to DNA copies. After that, the PCR product is prepared for capiller electrophoresis. As a result, base calling signals (trace files) are obtained from the bases of DNA, namely Adenine (A), Cytosine (C), Guanine (G), and Thymine (T) which are labeled with four different fluorescent dyes. A different analysis (i.e., mutation analysis, identification of subtypes of a virus known as a genotyping process and determination of species) can be accomplished from the results of a chromatogram that includes related sequences for the specific purpose.

**Figure 1:**
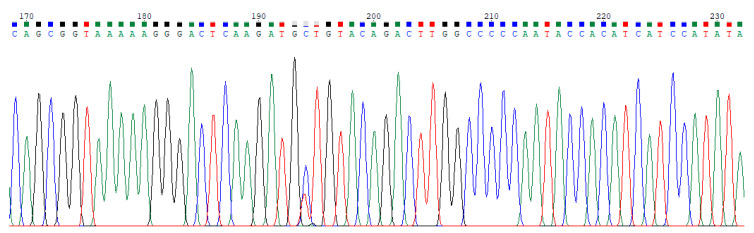
A sample of Hepatitis B Virus (HBV) trace file.

**Figure 2:**
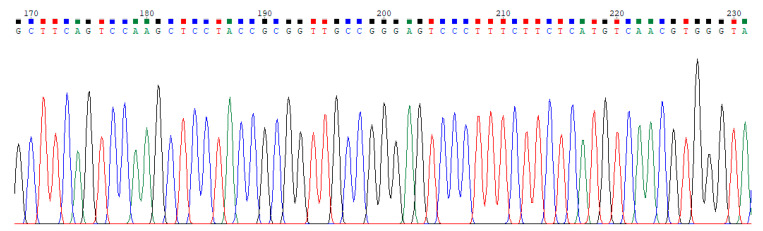
A sample of Hepatitis C Virus (HCV) trace file.

Sequential data modeling for the purpose of discriminating and classifying DNA chromatograms becomes very popular with the rapid development of sequencing techniques in molecular genetics and bioinformatics [1,2,3,4]. While some types of chromatograms can be manually recognized by an expert, it is hard to classify many of them without using any special software. Hepatitis B Virus (HBV) and Hepatitis C Virus (HCV) base calling signals are two types of hepatitis DNA chromatograms, and distinguishing these signals visually is impossible. Therefore, classification of hepatitis DNA trace files is an important issue in utilizing resources efficiently. The illustrations of HBV and HCV trace file samples are given in Figure 1 and Figure 2, respectively. These figures show the peaks of bases A, C, G, and T with different colors.

This study deals with classification of HBV and HCV trace files with support vector machines (SVM) using statistical and entropy-based feature extraction methods. Trace files are also accepted in a time-series manner and exhibit complex characteristics. In order to measure the complexity, approximate entropy (ApEn) was suggested by Pincus with the application of an electroencephalogram (EEG) series [5]. ApEn depends on the length of series and it takes a lower value than expected when the length is short. Since the sample entropy (SampEn) proposed by [6] is not affected by the length, it is more consistent than ApEn [7]. In addition, the calculation of SampEn is easier than ApEn. Permutation entropy (PE) [8] estimates the complexity of non-stationary, noisy and non-linear series by comparing neighboring values. These traditional entropy measures have been utilized for different purposes, especially in fault diagnosis and vibroarthographic (VAG) and electroencephalogram (EEG) signal-processing studies. However, none of these entropy measures are applicable for the systems which show structures on multiple spatial and temporal scales. In order to estimate multiscale complexity, multiscale entropy (MSE) was first suggested by Costa, Gollberg and Peng for the physiologic time series data [9]. The superiority of MSE was then showed by different time series data such as cardiac inter-beat (RR) [10,11] and human gait [12]. MSE uses single scale SampEn in order to quantify the complexity of coarse-grained series and different studies showed that it has some limitations based on the characteristics (e.g., existence of outliers, stationary) and length of the series [13,14,15]. A modification of MSE, namely multiscale permutation entropy (MPE), uses PE instead of SampEn, and the procedure is more robust to artifacts and observational noise in the time series data [16]. Except for techniques based on statistical theory, various researchers have offered suggestions with regard to using single and multiscale entropy measures as a feature extraction technique for classification of sequential data. While some studies investigate the performance of different sophisticated classifiers with extracted features using ApEn, SampEn and/or PE [17,18,19,20,21,22], others handle multiscale-based technique such as MPE [23,24]. These entropy measures have been used in biological time series data for the purpose of both quantifying complexity and the extraction of features in classification. However, there is no work available that uses entropy-based feature extraction methods for DNA trace files, especially for hepatitis DNA trace files. On the other hand, sophisticated classifiers within the concept of machine learning have been investigated in terms of their classification ability in the studies of DNA sequencing [25,26,27,28]. However, SVM [29,30] has been reported as a powerful classification tool compared with other supervised algorithms in recent years [31], and to the best our knowledge, none of the hepatitis DNA studies have examined SVM as a classifier.

In this study, a new framework for the classification of HBV and HCV trace files based on features extracted from four bases (i.e., A, C, G, D) of hepatitis DNA chromatograms is presented. Statistical-based and entropy-based features are extracted from the hepatitis DNA trace files. By using a statistical-based feature extraction method, it is intended to capture the statistical properties of four bases belonging to HBV and HCV with computing the values of mean, median and standard deviation. On the other hand, an entropy-based feature extraction method based on PE and MPE is utilized for the purpose of quantifying the complexity of these bases. Therefore, 24 computationally efficient features are extracted and later their different combinations are fed to SVM with different kernel functions such as linear, polynomial (Poly.) and radial bases (RBF). 

The rest of this study is organized as follows. Section 2 includes materials and methods of the study. The proposed framework is also given in this section. Model comparison results are presented in Section 3. A discussion and some concluding remarks are provided in Section 4 and Section 5, respectively.

## 2. Material and Methods

### 2.1. Dataset

**Figure 3:**
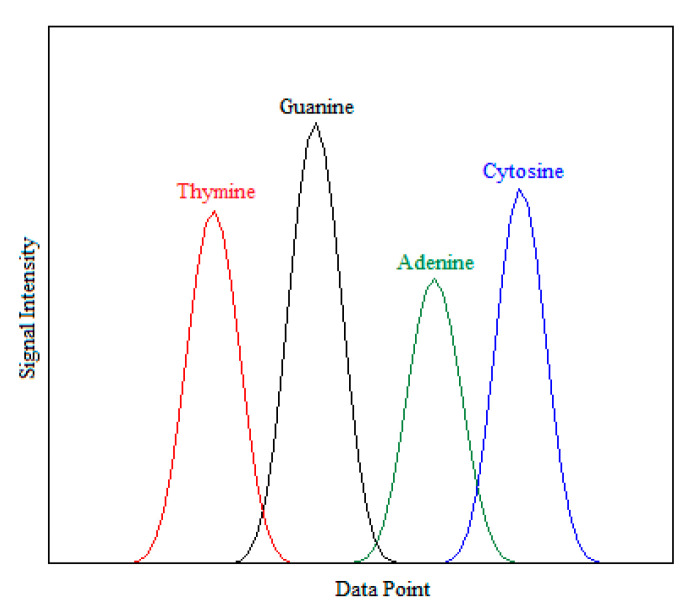
A sample of HBV trace file.

Hepatitis DNA trace files are obtained by “Phred” [32], which is widely used in academic and commercial laboratories as a base-calling software, embedded in a ABI-3730 capillary sequencer device (Applied Biosystems, Foster City, CA, USA) for DNA sequence traces. The data consists of 200 trace files, of which 96 are HBV and 104 are HCV. Type of hepatitis is taken as the dependent variable of constructed SVM models, which has a binary form. Therefore, hepatitis type is labeled as +1 if the trace file represents HBV, otherwise it is labeled as –1. Each trace file consists of four base calling signals time series shaped like Gaussian peaks (A, C, G, T bases). A typical segment of a DNA trace file is illustrated in Figure 3. Each base calling signal in the trace file is converted to an array using the “scfread” function of MATLAB 2017a software [33].

### 2.2. Feature Extraction

Identifying features extracted from the raw data correctly plays a vital role in the purpose of achieving better classification. Since the intensities of four base calling signals are different from each other, the raw data include trace files which cannot be directly used as an input for the classification process. For this reason, raw data should be converted to a mathematical representation which gives constant values. Different methods can be used in order to represent the raw data. Two types of extraction methods for arrays obtained from hepatitis DNA trace files are introduced in this study: (1) statistical-based feature extraction and (2) entropy-based feature extraction. Following subsections which provide the formulations of how features are extracted from a given base calling signal based on statistical and entropy theory. All calculations are carried out using MATLAB 2017a software [33]. 

#### 2.2.1. Statistical-Based Feature Extraction Method

Three statistical features based on descriptive statistical theory, including central tendency measures (mean and median) and a central dispersion measure (standard deviation), are used. These are frequently used statistics that reflect the property of DNA trace files [26,27].

Let N denote the length of each base-calling signal. The data points (located on X-axis in Figure 3) corresponding to signal intensities (located on Y-axis in Figure 3) for base calling signals A, C, G, and T can be expressed as $y_{A}\left(1,2,\ldots ,N\right)$, $y_{C}\left(1,2,\ldots ,N\right)$, $y_{G}=\left(1,2,\ldots ,N\right)$, and $y_{T}=\left(1,2,\ldots ,N\right)$, respectively. The mean and standard deviation formulas for each base calling signal are given as follows where j=A, C, G, T:

(1) $$\mu_{j}=\frac{1}{N}\overset{N}{\underset{i=1}{\sum }}y_{j}\left(i\right)$$

(2) $$\sigma_{j}={\left(\frac{1}{N}\overset{N}{\underset{i=1}{\sum }}{\left(y_{j}\left(i\right)-\mu_{j}\right)}^{2}\right)}^{\frac{1}{2}}$$

The intensities of base calling signal j are ordered and then the middle value is found by Equation (3) as $median_{j}$, where j=A, C, G, T:

(3) $$Prob\left(y_{j}\left(i\right)\leq median_{j}\right)=Prob\left(y_{j}\left(i\right)\geq median_{j}\right)=\frac{1}{2}$$

#### 2.2.2. Entropy-Based Feature Extraction Method

Two entropy-based feature extraction methods including PE and MPE are given in this section. The procedures of obtaining PE and MPE for a given base calling signal are presented below. 

● Permutation Entropy

The procedure of measuring PE of a given time series is a process of calculating Shannon entropy (ShEn) with mapping the original series to ordinal patterns. Using ordinal patterns has numerous advantages from different aspects [34]. For a given base calling signal j (j=A, C, G, T), the intensities, which exhibit the characteristics of a time series $Y_{j}={\left\{y_{j}\left(i\right)\right\}}_{i=1,2,\ldots ,N\;}$ with length N, m-dimensional vector, can be expressed as:

(4) $$y_{j}\left(i\right)=\left\{y_{j}\left(i\right),\;y_{j}\left(i+\tau \right),\ldots ,y_{j}\left(i+\left(m-1\right)\tau \right)\right\}$$

where the embedding dimension is denoted by m (≥2), and time lag is denoted by τ (ϵ ℕ). Here, $y_{j}\left(i\right)$ denotes overlapping segments with length m. According to parameter m, the number of possible permutations will be m! with permutation patterns $\pi_{p}$ where p=1,2,…,m!. For each $y_{j}\left(i\right)$, Equation (4) can be arranged in ascending order such that:

(5) $$y_{j}\left(i+\left(r_{1}-1\right)\tau \right)\leq \;y_{j}\left(i+\left(r_{2}-1\right)\tau \right)\leq \ldots \leq \;y_{j}\left(i+\left(r_{m}-1\right)\tau \right)$$

where 1 $\leq r_{i}\leq m$. Let the probability distribution for each permutation pattern π be shown with $P\left(\pi_{1}\right),\;P\left(\pi_{2}\right),\ldots ,P\left(\pi_{k}\right)$ where k≤m! and satisfy the condition $\sum_{l=1}^{k}\;P\left(\pi_{l}\right)=1$. Based on the ShEn, the PE of order m is now obtained as:

(6) $$HPE_{j}\left(m\right)=-\underset{\left\{\pi \right\}}{\sum }P\left(\pi_{l}\right)ln\left(P\left(\pi_{l}\right)\right)$$

When the relative frequencies of all permutation patterns are equal, the probabilities take the value of $\frac{1}{m!}$, and the maximum value for $HPE_{j}\left(m\right)$ is obtained as ln(m!) [35,36]. To make $HPE_{j}\left(m\right)$ scale-independent and comparable among different m, normalized PE $\left(HNPE_{j}\in \left[0,1\right]\right)$ is calculated by the following equation:

(7) $$HNPE_{j}=\frac{HPE_{j}\left(m\right)}{\;ln\left(m!\right)}$$

● Multiscale Permutation Entropy

The procedure of measuring MPE for a given intensity $Y_{j}={\left\{y_{j}\left(i\right)\;\right\}}_{i=1,2,\ldots ,N\;}$ of base calling signal j (j=A, C, G, T) with length N starts with creating a coarse-grained structure. The coarse-grained method introduced by Costa, Goldberg and Peng divides the original time series into non-overlapping windows of increasing length s, also called scale parameter [9]. The z-th element of multiple coarse-grained time series is obtained by:

(8) $$c_{j,z}^{\left(s\right)}=\frac{1}{s}\overset{zs}{\underset{i=\left(z-1\right)s+1}{\sum }}y_{j}\left(i\right)$$

where $1\leq z\leq \frac{N}{s}$. Here, $\frac{N}{s}$ is the length of the constructed coarse-grained time series. After determining the multiple coarse-grained time series, SampEn is then calculated. Instead of SampEn, Aziz and Arif suggested using PE (given in Equations (5) and (6)) to calculate the complexity of each coarse-grained series $C_{j}={\left\{c_{j}\left(z\right)\right\}}_{z=1,2,\ldots ,m\;}$ with length m where m-dimensional embedded vector can be expressed as follows [16]:

(9) $$c_{j}\left(z\right)=\left\{c_{j}\left(z\right),\;c_{j}\left(z+\tau \right),\ldots ,c_{j}\left(z+\left(m-1\right)\tau \right)\right\}$$

It should be noted that $MPE_{j}$ reduces to $HNPE_{j}$ when the scale parameter is equal to 1. $HNPE_{j}$ and $MPE_{j}$ are the entropy values of base calling signals’ intensities and calculated for all j=A, C, G, T bases. These entropy measures are used as features that will be included into the SVM classification models. 

### 2.3. Support Vector Machines

Binary class SVM aims to find the most appropriate hyperplane that separates two classes. The training set X with n samples has the form:

(10) $$X=\left\{\left(x_{1},y_{1}\right),\ldots ,\left(x_{n},y_{n}\right),\;x_{i}\in \;R^{d},y_{i}\in \left\{-1,+1\right\}\right\}$$

where $x_{i}$ denotes the set of input vectors, and $y_{i}$ is the set of corresponding labels which has a binary form [37]. The purpose is to estimate the parameters w and b which define the optimal hyperplane obtained from decision function expressed as sign (f(x)). Here, f(x) is the discriminant function used as the seperating hyperplane and can be defined as follows:

(11) $$f\left(x\right)=w^{T}x_{i}+b,\;w\in R^{d}\;\mathrm{and}\;b\in R$$

where the following constraint should be satisfied for this hyperplane:

(12) $$y_{i}\left(w^{T}x_{i}+b\right)\geq +1,\;i=1,\ldots ,n$$

A quadratic optimization problem which has the objective function $min\frac{1}{2}\|w{\|}^{2}$ with linear constraints given in Equation (12) is defined in order to obtain a maximum margin band. Using Lagrangian multipliers and Karush-Kuhn-Tucker conditions, the following dual problem can be obtained:

(13) $$L_{d}=\overset{n}{\underset{i=1}{\sum }}\alpha_{i}-\frac{1}{2}\overset{n}{\underset{i=1}{\sum }}\overset{n}{\underset{j=1}{\sum }}\alpha_{i}\alpha_{j}y_{i}y_{j}x_{i}{}^{T}x_{j}\;s.t.\;\sum_{i=1}^{n}\alpha_{i}y_{i}=0\;\mathrm{and}\;\alpha_{i}\geq 0$$

where $x_{i}$ inputs are named as support vectors corresponding to $\alpha_{i}$’s, and the values of $\alpha_{i}$’s are found by using one of the quadratic optimization methods for Equation (13). After that, the unknown parameters w and b are determined (for more details, see [38]). The slack variable ($\xi_{i}$) is added to the problem in the case of linearly non-separable data. The value of $\xi_{i}$ represents the total number of misclassifications.

When the data is linearly separable, the linear SVM mentioned above is applied; otherwise non-linear SVM should be preferred. The non-linear SVM outperforms the linear SVM when the complex-structured time series has many features. In non-linear SVM, the inputs are transformed from nonlinear to linear space with a specific kernel function. The aim is to find the hyperplane with the highest margin in the new space where the transformation is successfully achieved by kernels [39]. In the problem, the penalty parameter of the error term is shown by C and the term of $C\sum_{i=1}^{n}\xi_{i}$ is added to the object function [40]. After the transformation process of inputs, a linear SVM problem can be formulated for the new space [41]. Also, depending on kernels, Equation (13) is revised as the new dual optimization problem for the non-linear SVM given below:

(14) $$L_{d}=\overset{n}{\underset{i=1}{\sum }}\alpha_{i}-\frac{1}{2}\overset{n}{\underset{i=1}{\sum }}\overset{n}{\underset{j=1}{\sum }}\alpha_{i}\alpha_{j}y_{i}y_{j}K\left(x_{i}{}^{T}x_{j}\right)$$

where $K\left(x_{i}{}^{T}x_{j}\right)=\phi {\left(x_{i}\right)}^{T}\phi \left(x_{i}\right)$ is the kernel function. Linear, RBF and Poly. kernels are frequently used in SVM, and the preferability of a kernel over the others is based on expert knowledge and data structure. Table 1 shows the formulations of kernels used in this study, and γ and d are the kernel parameters. While only C parameter can be tuned in linear SVM, γ and d can be tuned in addition to C in RBF and Poly. kernel SVM, respectively. 

### 2.4. Performance Evaluation 

Different measures are used in evaluating the performance of SVM models with different kernel functions. Most of these can be derived from a confusion matrix which is a 2 × 2 table that holds information about the predicted versus actual class of observation. A typical confusion matrix is given in Table 2:

In the confusion matrix, TP and TN denote the number of correctly classified HBV and HCV trace files, respectively. Sensitivity (Se), sometimes called the TP rate, indicates the proportion of correctly classified HBV trace files. Analogously, specificity (Sp), also called the TN rate, shows the proportion of correctly classified HCV trace files. Accuracy (Acc) gives the the proportion of overall trace files that are classified correctly. Kappa (κ) statistics is an important agreement measure in the process of assessing the discriminative power of the relevant SVM model. κ statistics lies in the range between [–1,+1] and the perfect classification between HBV and HCV trace files is achieved when κ is found as 1.

### 2.5. Proposed Framework

The experimental setup of the proposed framework is described in the following steps:

Step 1: Preparing Dataset and Extracting Features

Two hundred trace files belong to Hepatitis DNA are obtained with Phred software. Hepatitis types (96 traces for HBV and 104 traces for HCV) are labeled as +1 and –1 if the related trace represents HBV and HCV, respectively. In order to extract features for the classification process, all trace files that contain four base calling signals are converted to arrays. 

In total, 24 features are extracted by two different feature extraction methods, namely statistical-based and entropy-based for SVM classification. Twelve features are obtained in the concept of statistical-based extraction and given in Table 3 where $\mu_{j}$, $\sigma_{j}$ and $median_{j}$ denote mean, standard deviation and median of base calling signal j(=A, C, G, T) respectively. The remaining 12 features are extracted with the entropy-based method; four of them are with single scale PE and eight are with multiscale PE. $MPE_{J}^{\left(2\right)}$ and $MPE_{J}^{\left(3\right)}$ demonstrate the multiscale PE of base calling signal j with scale parameters s=2 and s=3, respectively. The higher values of s correlated well with those from the results with s=2 and s=3. For this reason, other values of s are not considered. In addition, since choosing the parameters of embedding dimension m and time lag τ is an important issue which depends on the structure of time series, Bandt and Pompe suggested using the values of m=3,4,…, 7 and τ=1 in performing PE and MPE [8]. Also, Nalband, Prince and Agrawal followed this suggestion, using the same values [19]. Thus, these parameters are chosen as m=3 and τ=1. All calculations are carried out using MATLAB 2017a software [33].

Step 2: Creating Training and Testing Dataset

Data is split into train and test sets by random selections with a ratio of 10%, 20%, 30%, 40%, and 50% for each built SVM model in the training process. The grid-search technique on the kernel parameters using 10-fold cross-validation is utilized for the purpose of potentially obtaining a good combination of hyper-parameter values that produce a high generalization performance. The optimal regularization parameter (i.e., C) and kernel functions parameters (i.e., γ and d) are searched with defined values: C = (0, 0.01, 0.05, 0.1, 0.25, 0.5, 0.75, 1, 1.25, 1.5, 1.75, 2, 5), γ = (0, 0.01, 0.05, 0.1, 0.25, 0.5, 0.75, 1), and d = (1, 2, 3, 4, 5). 

Step 3: Performing Classification Process and Evaluating Results

The classification process of hepatitis DNA trace files is performed by using SVM with three different kernel functions. In this step, features extracted with statistical-based methods are used at first separately, and then together. Likewise, classification is performed using PE (i.e., MPE at *s* = 1), MPE at s = 2 and MPE at s = 3 features separately, and then together. SVM models using the mentioned features are built for each splitting proportion. Then performance evaluation measures Acc, Se, Sp, and κ are obtained for training and testing pairs. In addition, the number of support vectors (nSV) generated by the training phase of the relevant SVM model is found. This process is run a total of 10 times in order to aviod the random selection process effect. Thus, the performance evaluation measures and nSVs are calculated 10 times for each model. $\bar{Acc}$, $\bar{Se}$, $\bar{Sp}$, $\bar{\kappa }$, and $\bar{nSV}$ denote the mean values of Acc, Se, Sp, κ, and nSV, respectively. When training and testing errors are defined as $\varepsilon_{training}=1-{\bar{Acc}}_{training}$ and $\varepsilon_{testing}=1-{\bar{Acc}}_{testing}$, the error of the relevant SVM model is calculated by $\varepsilon_{diff}=\left|\varepsilon_{training}-\varepsilon_{testing}\right|$.

“Caret” and “Kernlab” libraries in R studio (version 1.2.1335, RStudio, Inc., Boston, MA, USA) programming language [42,43] are used in step 2 and step 3.

## 3. Results

### 3.1. Classification with Statistical-Based Features

Table 4 reports the classification performance of SVM models using statistical-based features for 10%, 20%, 30%, 40%, and 50% training sets and their corresponding testing sets.

When the statistical-based features are taken into account for 10%, 20%, 30%, 40%, and 50% training samples, the SVM-RBF kernel classifier with mean and all statistics features produces better classification performances in terms of both $\bar{Acc}$ and $\bar{\kappa }$. Additionally, the SVM models built with mean and all statistics features in all proportions of training samples indicate high classification accuracies ranging from nearly 93% to 99%. All the SVM models (with linear, Poly. and RBF kernels) for each training sample using median have the lowest classification performances among other statistical-based features. When the difference of error value between training and testing sets approaches to zero, it may be indicated that the model is not suffering from the over-fitting problem. The last column of Table 4 provides $\varepsilon_{diff}$, and these values are close to zero in general. On the other hand, Han and Jiang pointed out that the over-fitting problem in classification can be detected by using the expected values of sensitivity and specifity [44]. When these values are complementary, it can be said that the model has an over-fitting problem. It is shown in Table 4 that $\bar{Se}$ and $\bar{Sp}$ take on non-complementary values.

### 3.2. Classification with Entropy-Based Features

The classification performance of SVM models with entropy-based features for 10%, 20%, 30%, 40%, and 50% training sets and their corresponding testing sets are given in Table 5.

For 10% training samples, SVM-RBF kernel classifier with features of MPE at *s* = 2 has the highest performance in terms of $\bar{Acc}$ (95.6%) and $\bar{\kappa }$ (0.911). For the same training proportion, SVM-RBF kernel classifier with MPE at *s* = 3 and all entropies have the same values of $\bar{Acc\;}$= 95.5% and $\bar{\kappa }\;$= 0.909. In the case 20% and 30% training, the highest values of $\bar{Acc}$ are obtained with SVM-Poly. kernel classifier that uses all entropies as 96.6% and 98.3%, respectively. Also, this classifier produces the highest value of $\bar{\kappa }$ for 20% and 30% training samples. Results for 40% training samples show that SVM-RBF kernel classifier using all entropy-based features achieves better classification performance in terms of $\bar{Acc}$ and $\bar{\kappa }$ (98.9% and 0.978, respectively). Besides, SVM-Poly. kernel classifier with all entropy-based features takes the highest values of $\bar{Acc}$ and $\bar{\kappa }$ (98.1% and 0.962, respectively) for 50% training samples. Additionally, SVM models using entropy-based features in all training proportions achieve substantial classification performances where accuracies are ranging from nearly 93% to 99%. According to $\varepsilon_{diff}$, $\bar{Se}$ and $\bar{Sp}$ values, it can be concluded that the over-fitting problem does not appear in SVM models for 10%, 20%, 30%, 40%, and 50% training samples. SVM models using entropy-based features indicate very low $\varepsilon_{diff}$, ranging from 0.000 and 0.050. 

## 4. Discussion 

The characteristic of sequential data exhibits a complex structure. Due to the difficulty of distinguishing this type of data visually, the classification of sequential data has attracted notable attention of researchers in different areas. Most recent studies dealt with the complexity of the system, and therefore, used various types of entropy to extract features from the raw data. Features which reflect the behaviour of data truthfully do not only reduce the dimensionality of space, but also improve the classification quality. 

Recent studies for biological systems offered novel approaches to extract features based on single and multiscale entropy measures in order to achieve high classification accuracy. Especially, extracted features from EEG signal-based entropy helps researchers in the early diagnosis of epilepsy, different types of sleep disorders, and brain-related disorders such as Alzheimers [45]. Acharya et al. [46] extracted features from EEG signals by using ApEn, SampEn, and Phase Entropies (S1 and S2) for the purpose of detecting epilepsy. After applying different machine learning classification algorithms, it was shown that fuzzy classifier produced better classification performance (98%) in terms of the performance measures used in the study. Collected EEG signals from the brain were also discriminated with various classifiers after the extraction process, including entropy-based methods (i.e., ApEn and SampEn) in another important study [47]. AverageShEn, Renyi’s (RE), ApEn, SampEn, and S1 and S2 entropies were utilized to extract features from focal and non-focal epilepsy EEG signals in the study of Sharma, Pachori and Acharya [48]. It was reported that the least squares SVM with Morlet wavelet kernel function reached an 87% accuracy rate in classifying signals. For the classification of focal and non-focal EEG, Arunkumar et al. [49] proposed a methodology based on ApEn, SampEn and RE. Extracted features were fed into different classifiers such as NaïveBayes (NBC), SVM, k-nearest neighborhood (KNN), and non-nested generalized exemplars (NNGe). The results demonstrated that NNGe has the best classification performance with 98% accuracy. Also, a review about entropy-based feature extraction methods was presented for the diagnosis of epilepsy in [50]. To detect epileptic seizures, MSE was utilized as the feature extraction method, and SVM classifier was performed in [51]. The classification accuracies in classifying seizure, seizure-free and normal EEG signals were found to be higher than 98%. In sleep scoring classification, features were extracted from EEG signals using MSE, and SVM-based classifiers were performed in [52]. The overall accuracy rate was found to be 91.4%. To make accurate classifications of sleep stages, Rodríguez-Sotelo et al. [53] proposed a method based on J-means classification with EEG features extracted by fractal dimension, detrended fluctuation analysis, ShEn, ApEn, SampEn, and MSE. Extracted features were optimized with Q−α method, and then were fed to J-means classifier which achieved an average of 80% accuracy rate. Another important study which deals with sleep disorders was conducted by using 22 different EEG features including ApEn, SampEn and PE [54]. Extracted features were then fed to Wavelet transform and SVM classifiers. Recent studies have also showed that features extracted from entropy measures produce high classification performance in classifying human sleep EEG signals with different supervised and unsupervised machine learning methods [55,56,57]. To detect Alzheimer’s disease, various EEG features including entropy (e.g., ApEn, SampEn, PE) and statistical (e.g., mean, variance, standard deviation) measures were extracted in [58] and then fed to six classifiers including SVM, artificial neural network, KNN, NBC, and random forest. The proposed method indicated high classification accuracy ranging from nearly 89% to 97%.

Some of the important studies presented above can be seen as pioneers in classifying the sequential data obtained from biological systems and they demonstrated the usefulness of entropy-based feature extraction methods. On the other hand, an increasing number of studies in recent years have investigated the classification abilities of machine learning-based methods for genomic data [59,60]. Genomics is defined as one of the most important domains in bioinformatics [59] where computational methods need to be carefully utilized in order to discover useful but hidden information from biological systems. Extracting a set of features from the bases of DNA and then feeding to any supervised classifier for the purpose of labeling DNA trace files (e.g., high/low quality, genotyping of the viruses, species identification) is an important step to achieve high classification accuracy, as in all classification paradigms. To the best of our knowledge, there is no work which deals with entropy-based feature extraction methods for gene sequencing data. In this study, a new framework is proposed to classify hepatitis DNA trace files with SVM using extraction methods based on both statistics and entropy (i.e., PE and MPE) measures. The mathematical formulations of two extraction methods are introduced. The offered extraction methods are applied for the hepatitis DNA trace files and hence, the classification of the files as HBV and HCV is performed via SVM with three different kernel functions.

SVM models built with median features have low accuracies compared to models with other statistical-based features. In general, SVM-RBF kernel classifier using mean and all statistics features outperforms SVM models with other statistical-based features. On the other hand, SVM-RBF or SVM-Poly. kernel classifiers using all entropies achieve higher classification performances than SVM-linear classifier for all training samples except 10%. SVM models using both statistical and entropy-based features exhibit very close classification performances in terms of accuracies. 

When the best-performing SVM models for each training proportions are compared, it is found that the models with entropy-based features produce lower nSVs than models with statistical-based features and consequently yield lower complexity in the decision process.

According to Table 5, SVM-RBF kernel classifiers with entropy-based features have a higher percentage of nSVs compared with SVM-linear and SVM-Poly. kernel classifiers for all training proportions. Therefore, one can conclude that an over-fitting problem can appear. On the contrary, for each training proportion, it is found that $\varepsilon_{\mathrm{diff}}$ values are close to 0, $\bar{\mathrm{Se}}$ values are close to 1, and $\bar{\mathrm{Sp}}$ values are above 0.90. In addition, $\bar{\mathrm{Se}}$ and $\bar{\mathrm{Sp}}$ do not take complementary values. Thus, according to these values, it is not expected that an over-fitting problem can arise. On the other hand, SVM models using all entropies have lower nSVs compared with models using PE, MPE at *s* = 2 and MPE at *s* = 3 separately for training proportions from 30% to 50%. Thus, it can be concluded that less parameters are enough to define hyperplanes of problem complexity in the situation of SVM using all entropies. Moreover, cross-validation utilized in the training phase also contributes to overcome the over-fitting problem.

## 5. Conclusions 

The results demonstrate that the proposed framework produces remarkable classification performances based on both statistical and entropy features. By integrating this framework into the DNA sequencing devices, autonomous classification of DNA trace files, especially hepatitis DNA trace files that cannot be distinguished visually, can be achieved successfully. 

The proposed framework, which offers two different feature extraction methods, demonstrates that SVM models with statistical-based features have high performance as well as models with entropy-based features. Hence, it is suggested that entropies can be effectively used in the extraction of features from DNA trace files which produce non-stationary, noisy and non-linear signals. This feature extraction method can be considered either alone or combined with other extraction methods with the purpose of obtaining higher classification performance.

Although this study is designed for the classification of two class trace files (HBV and HCV), further studies can be concentrated on multi-class trace files such as the genotypes (sub-types) of hepatitis, other viruses and bacteria DNA trace files. Also, different supervised machine learning methods can be implemented and compared in terms of their classification ability.

## Figures and Tables

**Table 1:** Kernel functions.

Kernel	$K\left(x_{i}{}^{T}x_{j}\right)$
Linear	$x_{i}{}^{T}x_{j}$
Radial basis function	$\exp\left(-{\gamma \|x}_{i}-x_{j}{\|}^{2}\right),\gamma >0$
Polynomial	${\left(x_{i}{}^{T}x_{j}+1\right)}^{d},\gamma >0$

**Table 2:** Confusion matrix.

		Classifier Prediction Value	
		Positive	Negative
**Actual Value**	**Positive**	True positives (TP)	False negatives (FN)
	**Negative**	False positives (FP)	True negatives (TN)

**Table 3:** Feature Descriptions.

Feature		Base Calling Signal			
Method	Description	Adenine	Cytosine	Guanine	Thymine
Statistical Based	Mean	$\mu_{A}$	$\mu_{C}$	$\mu_{G}$	$\mu_{T}$
	Median	${\mathrm{median}}_{A}$	${\mathrm{median}}_{C}$	${\mathrm{median}}_{G}$	${\mathrm{median}}_{T}$
	Standard Deviation	$\sigma_{A}$	$\sigma_{C}$	$\sigma_{G}$	$\sigma_{T}$
Entropy Based	PE	${\mathrm{HNPE}}_{A}$	${\mathrm{HNPE}}_{C}$	${\mathrm{HNPE}}_{G}$	${\mathrm{HNPE}}_{T}$
	MPE with *s* = 2	${\mathrm{MPE}}_{A}^{\left(2\right)}$	${\mathrm{MPE}}_{C}^{\left(2\right)}$	${\mathrm{MPE}}_{G}^{\left(2\right)}$	${\mathrm{MPE}}_{T}^{\left(2\right)}$
	MPE with *s* = 3	${\mathrm{MPE}}_{A}^{\left(3\right)}$	${\mathrm{MPE}}_{C}^{\left(3\right)}$	${\mathrm{MPE}}_{G}^{\left(3\right)}$	${\mathrm{MPE}}_{T}^{\left(3\right)}$

**Table 4:** Overall performance measures of classifications using statistical-based features.

Feature	SVM	Training (10%)					Testing				$\varepsilon_{diff}$
		$\bar{Acc}$	$\bar{\kappa }$	$\bar{Se}$	$\bar{Sp}$	$\bar{nSV}$	$\bar{Acc}$	$\bar{\kappa }$	$\bar{Se}$	$\bar{Sp}$	
Mean	Linear	0.983	0.966	0.969	1.000	10.9	0.961	0.923	0.927	0.999	0.022
	Poly. Kernel	0.970	0.940	0.945	1.000	11.8	0.960	0.921	0.924	1.000	0.010
	RBF Kernel	0.995	0.989	0.992	1.000	15.3	0.980	0.960	0.987	0.973	0.015
Median	Linear	0.793	0.532	0.669	0.861	12.9	0.708	0.425	0.590	0.844	0.085
	Poly. Kernel	0.905	0.782	0.761	1.000	10.2	0.772	0.555	0.637	0.928	0.133
	RBF Kernel	0.825	0.618	0.740	0.891	17.6	0.718	0.442	0.613	0.836	0.107
Standard Deviation	Linear	0.958	0.902	0.937	0.969	10.7	0.903	0.809	0.854	0.958	0.055
	Poly. Kernel	0.980	0.957	0.979	0.974	8.9	0.922	0.846	0.871	0.979	0.058
	RBF Kernel	0.970	0.932	0.972	0.958	15.1	0.963	0.927	0.967	0.960	0.007
All Statistics	Linear	0.992	0.984	0.985	0.999	8.9	0.953	0.906	0.913	0.996	0.039
	Poly. Kernel	0.985	0.969	0.975	1.000	10.7	0.938	0.878	0.885	0.996	0.047
	RBF Kernel	0.990	0.979	1.000	0.977	15.5	0.972	0.945	0.994	0.948	0.018
		**Training (20%)**					**Testing**				$\varepsilon_{\mathrm{diff}}$
Mean	Linear	0.981	0.961	0.963	1.000	18.4	0.967	0.935	0.938	0.999	0.014
	Poly. Kernel	0.997	0.995	0.995	1.000	19.4	0.964	0.928	0.934	0.996	0.033
	RBF Kernel	0.995	0.989	0.991	1.000	30.4	0.983	0.966	0.990	0.975	0.012
Median	Linear	0.801	0.604	0.621	0.991	22.5	0.770	0.547	0.585	0.971	0.031
	Poly. Kernel	0.905	0.796	0.892	0.890	19.2	0.834	0.671	0.812	0.863	0.071
	RBF Kernel	0.832	0.661	0.674	0.982	27.2	0.773	0.554	0.620	0.945	0.059
Standard Deviation	Linear	0.955	0.908	0.932	0.978	17.7	0.931	0.863	0.884	0.982	0.024
	Poly. Kernel	0.987	0.973	1.000	0.971	12.7	0.947	0.895	0.926	0.970	0.040
	RBF Kernel	0.990	0.979	0.993	0.985	26.0	0.964	0.928	0.976	0.951	0.026
All Statistics	Linear	0.989	0.978	0.979	1.000	17.0	0.970	0.941	0.945	0.998	0.019
	Poly. Kernel	0.990	0.979	0.979	1.000	16.3	0.970	0.941	0.945	0.998	0.020
	RBF Kernel	0.997	0.994	1.000	0.993	30.1	0.975	0.950	0.995	0.953	0.022
		**Training (30%)**					**Testing**				$\varepsilon_{\mathrm{diff}}$
Mean	Linear	0.986	0.972	0.974	1.000	22.7	0.968	0.937	0.939	1.000	0.018
	Poly. Kernel	0.995	0.989	0.990	1.000	16.8	0.976	0.952	0.958	0.995	0.019
	RBF Kernel	0.991	0.983	0.983	1.000	43.2	0.984	0.968	0.991	0.976	0.007
Median	Linear	0.807	0.617	0.638	0.989	31.0	0.784	0.574	0.608	0.976	0.023
	Poly. Kernel	0.921	0.839	0.897	0.934	20.4	0.839	0.681	0.809	0.879	0.082
	RBF Kernel	0.846	0.696	0.719	0.990	36.6	0.764	0.533	0.564	0.977	0.082
Standard Deviation	Linear	0.954	0.908	0.925	0.984	25.5	0.936	0.872	0.897	0.978	0.018
	Poly. Kernel	0.988	0.975	0.993	0.980	14.6	0.955	0.909	0.947	0.963	0.033
	RBF Kernel	0.985	0.969	0.987	0.982	31.6	0.972	0.945	0.986	0.958	0.013
All Statistics	Linear	0.990	0.980	0.981	1.000	21.3	0.976	0.952	0.954	0.999	0.014
	Poly. Kernel	0.995	0.989	0.989	1.000	22.1	0.972	0.945	0.952	0.995	0.023
	RBF Kernel	0.996	0.993	0.996	0.996	41.5	0.980	0.959	0.995	0.962	0.016
		**Training (40%)**					**Testing**				$\varepsilon_{\mathrm{diff}}$
Mean	Linear	0.984	0.969	0.970	1.000	25.0	0.970	0.941	0.944	1.000	0.014
	Poly. Kernel	0.990	0.979	0.985	0.994	29.1	0.980	0.960	0.963	0.998	0.010
	RBF Kernel	0.993	0.987	0.987	1.000	57.3	0.990	0.979	0.991	0.987	0.003
Median	Linear	0.818	0.636	0.651	0.990	38.5	0.789	0.586	0.624	0.975	0.029
	Poly. Kernel	0.916	0.829	0.931	0.894	29.0	0.840	0.682	0.855	0.829	0.076
	RBF Kernel	0.828	0.661	0.684	0.989	48.0	0.827	0.656	0.678	0.984	0.001
Standard Deviation	Linear	0.954	0.907	0.923	0.987	33.2	0.932	0.865	0.890	0.979	0.022
	Poly. Kernel	0.995	0.989	0.997	0.991	17.1	0.968	0.936	0.971	0.964	0.027
	RBF Kernel	0.986	0.972	0.989	0.981	33.1	0.974	0.948	0.982	0.965	0.012
All Statistics	Linear	0.992	0.984	0.985	1.000	23.8	0.975	0.951	0.954	0.999	0.017
	Poly. Kernel	0.995	0.989	0.990	1.000	21.0	0.981	0.963	0.967	0.996	0.014
	RBF Kernel	0.996	0.992	0.995	0.997	41.5	0.984	0.968	0.983	0.984	0.012
		**Training (50%)**					**Testing**				$\varepsilon_{\mathrm{diff}}$
Mean	Linear	0.987	0.974	0.975	1.000	25.4	0.971	0.941	0.943	1.000	0.016
	Poly. Kernel	0.992	0.983	0.986	0.998	29.8	0.980	0.959	0.968	0.993	0.012
	RBF Kernel	0.993	0.985	0.990	0.995	73.4	0.989	0.977	0.988	0.988	0.004
Median	Linear	0.817	0.639	0.655	0.995	47.8	0.803	0.611	0.632	0.987	0.014
	Poly. Kernel	0.913	0.823	0.959	0.857	34.4	0.867	0.735	0.900	0.837	0.046
	RBF Kernel	0.841	0.679	0.677	1.000	54.4	0.802	0.614	0.643	0.992	0.039
Standard Deviation	Linear	0.949	0.898	0.919	0.981	41.1	0.937	0.874	0.900	0.977	0.012
	Poly. Kernel	0.984	0.967	0.990	0.976	32.2	0.970	0.939	0.965	0.974	0.014
	RBF Kernel	0.987	0.973	0.995	0.977	35.7	0.968	0.935	0.977	0.954	0.019
All Statistics	Linear	0.995	0.990	0.991	1.000	21.3	0.980	0.961	0.963	0.999	0.015
	Poly. Kernel	0.998	0.995	0.996	1.000	18.0	0.979	0.957	0.966	0.992	0.019
	RBF Kernel	0.996	0.991	0.996	0.995	37.8	0.991	0.981	0.989	0.990	0.005

**Table 5:** Overall performance measures of classifications using entropy-based features.

Feature	SVM	Training (10%)					Testing				$\varepsilon_{diff}$
		$\bar{Acc}$	$\bar{\kappa }$	$\bar{Se}$	$\bar{Sp}$	$\bar{nSV}$	$\bar{Acc}$	$\bar{\kappa }$	$\bar{Se}$	$\bar{Sp}$	
PE	Linear	0.944	0.880	0.984	0.894	10.8	0.933	0.867	0.957	0.909	0.011
	Poly. Kernel	0.960	0.919	1.000	0.921	8.4	0.950	0.900	0.977	0.921	0.010
	RBF Kernel	0.965	0.928	1.000	0.931	16.1	0.950	0.900	0.994	0.902	0.015
MPE with *s* = 2	Linear	0.954	0.904	0.995	0.911	10.8	0.941	0.882	0.973	0.905	0.013
	Poly. Kernel	0.995	0.990	1.000	0.990	9.7	0.945	0.890	0.954	0.935	0.050
	RBF Kernel	0.945	0.890	1.000	0.894	15.5	0.956	0.911	1.000	0.909	0.011
MPE with *s* = 3	Linear	0.949	0.894	0.984	0.909	11.3	0.937	0.874	0.963	0.909	0.012
	Poly. Kernel	0.980	0.959	1.000	0.958	8.5	0.935	0.871	0.934	0.937	0.045
	RBF Kernel	0.955	0.905	1.000	0.892	15.6	0.955	0.909	1.000	0.907	0.000
All Entropies	Linear	0.954	0.903	0.987	0.915	10.1	0.945	0.890	0.981	0.905	0.009
	Poly. Kernel	0.980	0.956	1.000	0.950	11.0	0.954	0.908	0.987	0.919	0.026
	RBF Kernel	0.970	0.938	1.000	0.940	15.7	0.955	0.909	0.994	0.911	0.015
		**Training (20%)**					**Testing**				$\varepsilon_{diff}$
PE	Linear	0.945	0.889	0.990	0.897	20.6	0.949	0.899	0.988	0.908	0.004
	Poly. Kernel	0.980	0.959	1.000	0.956	12.7	0.946	0.893	0.953	0.940	0.034
	RBF Kernel	0.955	0.906	1.000	0.896	31.4	0.953	0.907	1.000	0.905	0.002
MPE with *s* = 2	Linear	0.946	0.889	0.992	0.894	21.2	0.950	0.901	0.989	0.909	0.004
	Poly. Kernel	0.977	0.954	0.995	0.958	16.1	0.947	0.894	0.989	0.901	0.030
	RBF Kernel	0.955	0.909	1.000	0.907	32.9	0.955	0.909	1.000	0.906	0.000
MPE with *s* = 3	Linear	0.948	0.894	0.992	0.904	20.4	0.950	0.900	0.989	0.907	0.002
	Poly. Kernel	0.970	0.938	0.991	0.952	16.9	0.949	0.897	0.982	0.912	0.021
	RBF Kernel	0.965	0.928	0.993	0.933	31.2	0.941	0.883	0.972	0.910	0.024
All Entropies	Linear	0.952	0.902	0.989	0.911	19.8	0.950	0.900	0.988	0.909	0.002
	Poly. Kernel	0.987	0.971	1.000	0.964	10.6	0.966	0.932	0.977	0.955	0.021
	RBF Kernel	0.975	0.948	1.000	0.947	34.4	0.950	0.899	0.995	0.901	0.025
		**Training (30%)**					**Testing**				$\varepsilon_{diff}$
PE	Linear	0.949	0.897	0.989	0.906	29.4	0.948	0.896	0.987	0.906	0.001
	Poly. Kernel	0.986	0.973	1.000	0.972	17.5	0.957	0.915	0.970	0.944	0.029
	RBF Kernel	0.956	0.911	1.000	0.905	49.9	0.954	0.908	1.000	0.906	0.002
MPE with *s* = 2	Linear	0.953	0.905	0.993	0.909	28.6	0.949	0.897	0.990	0.905	0.004
	Poly. Kernel	0.996	0.993	0.996	0.995	11.7	0.964	0.928	0.970	0.959	0.032
	RBF Kernel	0.950	0.896	1.000	0.888	50.2	0.956	0.912	1.000	0.911	0.006
MPE with *s* = 3	Linear	0.950	0.899	0.993	0.904	30.7	0.950	0.899	0.989	0.907	0.000
	Poly. Kernel	0.981	0.962	0.989	0.972	15.8	0.937	0.874	0.918	0.961	0.044
	RBF Kernel	0.963	0.924	1.000	0.917	50.0	0.951	0.902	1.000	0.901	0.012
All Entropies	Linear	0.960	0.920	0.991	0.928	28.2	0.948	0.896	0.990	0.901	0.012
	Poly. Kernel	0.996	0.996	1.000	0.992	10.6	0.983	0.967	0.983	0.983	0.013
	RBF Kernel	0.983	0.965	1.000	0.963	36.1	0.970	0.939	1.000	0.937	0.013
		**Training (40%)**					**Testing**				$\varepsilon_{diff}$
PE	Linear	0.951	0.901	0.989	0.909	39.0	0.947	0.893	0.987	0.903	0.004
	Poly. Kernel	0.990	0.979	0.995	0.982	13.5	0.969	0.938	0.968	0.970	0.021
	RBF Kernel	0.948	0.896	1.000	0.891	70.0	0.959	0.917	1.000	0.914	0.011
MPE with *s* = 2	Linear	0.951	0.901	0.994	0.905	37.4	0.950	0.899	0.989	0.906	0.001
	Poly. Kernel	0.996	0.992	1.000	0.991	14.6	0.961	0.923	0.960	0.963	0.035
	RBF Kernel	0.948	0.897	1.000	0.898	68.8	0.959	0.917	1.000	0.912	0.011
MPE with *s* = 3	Linear	0.949	0.896	0.991	0.903	40.7	0.951	0.901	0.990	0.908	0.002
	Poly. Kernel	0.976	0.951	0.995	0.952	21.3	0.952	0.904	0.971	0.933	0.024
	RBF Kernel	0.956	0.911	1.000	0.904	68.4	0.954	0.908	1.000	0.907	0.002
All Entropies	Linear	0.964	0.927	0.994	0.928	30.3	0.952	0.904	0.990	0.912	0.012
	Poly. Kernel	0.993	0.987	1.000	0.986	14.0	0.977	0.954	0.987	0.967	0.016
	RBF Kernel	0.996	0.992	1.000	0.991	29.3	0.989	0.978	1.000	0.977	0.007
		**Training (50%)**					**Testing**				$\varepsilon_{diff}$
PE	Linear	0.948	0.895	0.990	0.903	49.5	0.951	0.901	0.989	0.909	0.003
	Poly. Kernel	0.992	0.983	0.995	0.987	11.1	0.960	0.919	0.971	0.948	0.032
	RBF Kernel	0.959	0.917	1.000	0.916	76.6	0.955	0.908	1.000	0.903	0.004
MPE with *s* = 2	Linear	0.952	0.904	0.996	0.905	41.4	0.950	0.900	0.989	0.907	0.002
	Poly. Kernel	0.996	0.991	0.996	0.995	13.4	0.972	0.943	0.966	0.977	0.024
	RBF Kernel	0.947	0.893	1.000	0.893	84.4	0.963	0.924	1.000	0.919	0.016
MPE with *s* = 3	Linear	0.951	0.902	0.992	0.907	46.6	0.949	0.898	0.991	0.904	0.002
	Poly. Kernel	0.984	0.967	0.996	0.969	16.0	0.946	0.891	0.960	0.931	0.038
	RBF Kernel	0.954	0.907	1.000	0.903	86.4	0.956	0.911	1.000	0.909	0.002
All Entropies	Linear	0.966	0.932	0.994	0.935	30.8	0.956	0.912	0.991	0.919	0.010
	Poly. Kernel	0.999	0.997	1.000	0.997	14.6	0.981	0.962	0.991	0.971	0.018
	RBF Kernel	0.988	0.975	1.000	0.975	41.9	0.979	0.957	1.000	0.953	0.009
